# Molecular epidemiology of human respiratory syncytial virus among children in Japan during three seasons and hospitalization risk of genotype ON1

**DOI:** 10.1371/journal.pone.0192085

**Published:** 2018-01-29

**Authors:** Akinobu Hibino, Reiko Saito, Kiyosu Taniguchi, Hassan Zaraket, Yugo Shobugawa, Tamano Matsui, Hiroshi Suzuki, for the Japanese HRSV Collaborative Study Group

**Affiliations:** 1 Division of International Health (Public Health), Niigata University Graduate School of Medical and Dental Sciences, Niigata, Japan; 2 Pediatrics, National Mie Hospital, Mie, Japan; 3 Department of Pathology, Immunology, and Microbiology, Faculty of Medicine American University of Beirut, Beirut, Lebanon; 4 Center for Infectious Disease Research, Faculty of Medicine American University of Beirut, Beirut, Lebanon; 5 Infectious Disease Surveillance Center, National Institute of Infectious Diseases, Tokyo, Japan; 6 School of Nursing, Niigata Seiryo University, Niigata, Japan; University of Hong Kong, HONG KONG

## Abstract

We investigated the genetic diversity, the circulation patterns, and risk for hospital admission of human respiratory syncytial virus (HRSV) strains in Japan between 2012 through 2015. During the study period, 744 HRSV-positive cases were identified by rapid diagnostic test. Of these, 572 samples were positive by real-time PCR; 400 (69.9%) were HRSV-A, and 172 (30.1%) were HRSV-B. HRSV-A and -B alternated as the dominant strain in the subsequent seasons. Phylogenetic tree analysis of the second hyper-variable region of the G protein classified the HRSV-A specimens into NA1 (n = 242) and ON1 (n = 114) genotypes and the HRSV-B specimens into BA9 (n = 60), and BA10 (n = 27). The ON1 genotype, containing a 72-nucleotide duplication in the G protein’s second hyper-variable region, was first detected in the 2012–2013 season but it predominated and replaced the older NA1 HRSV-A in the 2014–2015 season, which also coincided with a record number of HRSV cases reported to the National Infectious Disease Surveillance in Japan. The risk of hospitalization was 6.9 times higher for the ON1 genotype compared to NA1. In conclusion, our data showed that the emergence and predominance of the relatively new ON1 genotype in Japan was associated with a record high number of cases and increased risk for hospitalization.

## Introduction

Human respiratory syncytial virus (HRSV) is a major cause of acute lower respiratory infection (ALRI) in infants and young children [1]. Importantly, HRSV accounts for a large number of hospitalization and mortality in respiratory virus infections of children under the age of 5 years worldwide [2,3]. Studies showed that almost all children get infected with HRSV at least one time by the age of 2 years [4], yet reinfections with HRSV are common throughout life [5].

HRSV belongs to the family *Paramyxoviridae*, non-segmented negative-sense single-stranded enveloped RNA viruses. HRSV has been classified into subgroups A and B (HRSV-A and HRSV-B) based on the genetic characteristics of the attachment protein (G protein) and reaction to monoclonal antibodies [6]. The F protein is conserved, while the G protein is the most variable HRSV protein [7]. The C-terminal region of G protein (the second hypervariable region, herein HVR2) is used as the basis for HRSV genotyping to study its evolution [8,9]. HRSV-A has been classified into genotypes such as GA1–7, SAA1–2, NA1–4, and ON1 [8–14] and HRSV-B into GB1–5, SAB1–4, URU1–2, BA1–12, and THB [8,9,15–21].

Recently, a novel HRSV-A ON1 genotype, with a 72-nucleotide duplication in HVR2, was identified in Canada in 2010 [12]. Genotype ON1 evolved from NA1that we previously reported as a new genotype in 2004–2005 [11] and rapidly spread to many countries after 2010 [22–25]. BA genotype, with a 60-nucleotide duplication in HVR2, was first reported in Buenos Aires in 1999 and became the predominant HRSV-B strain [17,26]. Recent study showed BA9 and BA10 that we reported [16] became the most prevalent HRSV-B strains globally [23,27–30].

According to the national HRSV surveillance in Japan (the Infectious Diseases Weekly Report released by National Institute of Infectious Diseases, Japan, http://www.niid.go.jp/niid/ja/idwr.html), the number of patients infected with HRSV in December 2014 reached a record high number of 30,000 cases. However, due to the lack of nationwide molecular studies of HRSV in Japan it was not determined whether this surge in HRSV cases is associated with the emergence of a new genotype.

Here we report the first nationwide molecular epidemiology study of HRSV strain that circulated in Japan during the 2012–2013 to 2014–2015 seasons. Furthermore, we evaluated risk of hospitalization by ON1 genotype compared to other HRSV genotypes that circulated at the same period.

## Materials and methods

### Study population and clinical samples

The study was conducted over three seasons from September 2012 to August 2015 at 18 pediatric outpatient clinics and hospitals in 17 out of 47 prefectures in Japan; Hokkaido, Aomori, Tokyo, Chiba, Kanagawa, Niigata, Shizuoka, Aichi, Mie, Shiga, Osaka, Hyogo, Kagawa, Yamaguchi, Fukuoka, Kumamoto, and Okinawa. Children under 5 years of age who visited the medical institutions with a sudden onset of symptoms such as wheezing, cough, rhinorrhea, or fever (≥37.5°C) were screened with a rapid diagnostic test (RDT). The HRSV RDT kits used were Quick-navi Flu+RSV (Denka Seiken, Tokyo, Japan), Quick-navi RSV (Denka Seiken, Tokyo, Japan), Primecheck RSV (Alfresa Pharma, Osaka, Japan), Rapid Testa RSV-Adeno (Sekisui Medical, Tokyo, Japan), ImunoAce RSV (Tauns, Shizuoka, Japan), and Check RSV (Meiji Seika Pharma, Tokyo, Japan). All these RDTs are covered by the national health insurance under 1 year old children at outpatient clinics and commonly used for the diagnostic purpose in Japan. Patients with a positive RDT were enrolled in this study upon obtaining a written informed consent from the parent or guardian. Nasopharyngeal aspirates or nasal swabs were then collected from the patients by the clinicians. Basic demographic and clinical data, such as age, gender, body temperature, date of onset, date of clinical first visit, premature birth (gestation week <36 weeks), low birth weight (<2,500 g), underlying conditions (e.g. congenital heart diseases, congenital chronic lung diseases, immune deficiencies, Down Syndrome, or asthma), and admission to hospital were recorded. This study was approved by the medical faculty ethics committee of Niigata University Medical School (acceptance No. 2020).

The samples were transported to the Division of International Health, Graduate School of Medical and Dental Sciences, Niigata University, and were kept frozen at -80°C until further examination.

### Real-time PCR for subgrouping

Viral RNA is extracted from 100-μl of the clinical specimens by using an Extragen II kit (Kainos, Tokyo, Japan) according to the manufacturer’s instructions. Reverse Transcription to create complementary DNA (cDNA) is performed using random primers and moloney murine leukemia virus reverse transcriptase (Invitrogen Corp. Carlsbad, CA) by incubation at 37°C for 1 hour.

The real-time PCR was conducted on a Thermal Cycler Dice Real Time PCR Systems TP800 machine (Takara Bio Inc., Shiga, Japan), using the Premix Taq^®^ (TaKaRa Taq^™^ Version 2.0, Takara Bio Inc). Screening for HRSV was performed using TaqMan probe real-time PCR method targeting the M protein (Table 1). Positive samples were further subgrouped using TaqMan probe sets targeting the F protein of HRSV (Table 1). We used F protein because the protein is genetically more stable than G protein that is normally used for subgrouping [7].

**Table 1:** Sequences of primers and probes used in this study.

Purpose	Target gene	Primer or probe name	Sequence (5’→3’)	Amplicon size (base pairs)
**HRSV Generic screening**	M protein	RSV-Forward	GCAAATATGGAAACATACGTGAACA	116
		RSV-Reverse	GCACCCATATTGTWAGTGATGCA	
		RSV-P	(FAM)-CTTCACgAAggCTCCACATACACAgCWg-(Eclipse)	
**HRSV-A subtyping**	F protein	RSV-A-Forward	ATCAGAAAAAGTTAATGTCCA	118
		RSV-A-Reverse	ACACCATATAGTGGTAATTGT	
		RSV-A-P	(FAM)-TCAAATAgTTAgACAgCAAAgTTACTCT-(BHQ1)	
**HRSV-B subtyping**	F protein	RSV-B-Forward	GTTTAACAAGGACTGATAGAG	153
		RSV-B-Reverse	TGTTACAAAGGCTGACTT	
		RSV-B-P	(FAM)-ACTGATCCTGCATTATCACARTACCA-(BHQ1)	
**HRSV-A genotyping**	G protein	GPA_RSV	GAAGTGTTCAACTTTGTACC	487
		F1	GGCAAATAACAATGGAGTTG	
**HRSV-B genotyping**	G protein	GPB_RSV	AAGATGATTACCATTTTGAAGT	507
		F1	GGCAAATAACAATGGAGTTG	

The real-time PCR assay was carried out in a 25-μl volume consisting of 12.5μl of 2X Premix Taq solution containing 1.25U of TaKaRa Taq DNA Polymerase, 0.25μl of 20 pmol/ml of each oligonucleotide primer, 1μl of 5 pmol/ml of TaqMan probe, 1 μl of cDNA template and 10 μl of nuclease free water. The target sequence amplification was conducted as follows: initial holding at 95°C for 10 sec, followed by 50 cycles of 95°C for 5sec, 60°C for 30 sec. Positive Ct values are set ≤ 35 cycles and the curves over 35 were considered as negative.

### Nucleotide sequencing and genotyping

After subgrouping by real-time PCR, positive samples underwent conventional PCR targeting at the glycoprotein (G) gene’s HVR2. The primers used for PCR are listed in Table 1. 1μl of viral cDNA is added to 20μl of the reaction mixtures. Thermal cycling conditions for the PCR are as follows: initial denaturation at 94 °C for 2min, followed by 30 cycles of 94°C for 1min, 50 °C for 1min, and 72 °C for 2 min, with a final 7 min of extension at 72 °C. Amplified PCR products is purified with primer removal kits (QIAquick PCR Purification Kit, QIAGEN, Inc.), labeled with a BigDye terminator (version 3.1) cycle sequencing kit (Applied Biosystems, Carlsbad, USA) according to the manufacturer’s instructions, and then analyzed on an ABI Prism 3130xl Genetic Analyzer (Thermo Fisher Scientific Inc, Waltham, USA). The PCR primers were used as the sequencing primers.

The obtained sequences were assembled using Lasergene SeqMan Pro package version 12.2.0 (DNASTAR, Madison, USA). Sequence alignments were performed using CLUSTALW in BioEdit software (http://www.mbio.ncsu.edu/BioEdit/). Phylogenetic trees of the G protein’s HVR2 were generated by the neighbor-joining method using the Maximum Composite Likelihood model for substitution model and complete deletion for gap or missing data treatment with MEGA version 6.0 software [31]. Bootstrap probabilities are calculated 1000 iterations to evaluate confidence estimates. We used the neighbor-joining trees generate by the Maximum Composite Likelihood model since the model is reported to be as accurate as the ones generated by the Maximum-Likelihood [32]. Related sequence data of HRSV-A and HRSV-B were searched with Basic Local Alignment Search Tool (BLAST). The returned sequences were downloaded from GenBank and included in the phylogenetic trees together with the sequences obtained in this study. Pairwise nucleotide distances (*p* distances) within and between clades for HRSV-A and HRSV-B were calculated by using the Kimura 2-parameter model with MEGA 6.0 [31]. A new clade designation was defined as a cluster of strains with bootstrap value of 70–100% [16] and a p-distance less than 0.049, a minimal threshold for sorting viruses into different genotypes proposed by Trento et al. [33].

### RSV infection surveillance

The monthly distribution of HRSV genotypes from this study and the HRSV cases reported to National Infectious Disease Surveillance was analyzed. For National Infectious Disease Surveillance, approximately 3,000 pediatric sentinels (hospitals and clinics) report the weekly number of patients diagnosed as HRSV infection to prefecture or municipal public health sectors in Japan. An HRSV case is defined by a positive RDT, virus isolation or antibody rise in paired sera according the Ministry of Health, Labor and Welfare guidelines (http://www.mhlw.go.jp/bunya/kenkou/kekkaku-kansenshou11/01-05-15.html). The prefectural data is reported to the Infectious Disease Surveillance Center in the National Institute of Infectious Disease (NIID), Tokyo, Japan. NIID releases the number of HRSV cases on a weekly basis through its website (http://idsc.nih.go.jp/idwr/index.html). A season is defined as the period between September of a given year through August in the subsequent year.

### Assessment of the hospitalization risk

Univariate and multivariate analyses were performed to evaluate the risk of hospitalization by genotype. Hospitalization was cross-tabulated with age groups (< 6 or ≥ 6 months), prematurity (gestation week <36 weeks) and/or low birth weight at birth (< 2,500 g), underlying conditions (e.g. congenital heart diseases, congenital chronic lung diseases, immune deficiencies, Dawn Syndrome, or asthma), and HRSV genotypes (NA1, ON1, BA9 or BA10). Chi-square test and Fisher’s exact test were used to compare proportions of 2 by 2 table, or 2 by multiple table. Logistic regression analysis was employed to assess the association between genotype (NA1, ON1, BA9 or BA10) and hospitalization adjusted by age, prematurity and/or low birth weight, underlying conditions, and HRSV genotypes. A *P* value less than 0.05 was considered as statistically significant. All statistical test was performed by EZR software (ver1.35)[34].

### Nucleotide sequences accession numbers in GenBank (DDBJ)

The nucleotide sequences of HRSV-A (360 strains) and HRSV-B (87 strains) were registered to the GenBank (DDBJ) under the accession numbers from LC037455 to LC037938 (S1 Table). Note that 4 strains (RSA/Shizuoka/13RS206-2/2014, RSA/Shizuoka/14RS073-3/2014, RSA/Shizuoka/14RS073-4/2014, RSA/Shizuoka/14RS074-3/2014) were collected repeatedly from the three patients (Shizuoka/13RS206, Shizuoka/14RS073, and Shizuoka/14RS074) at the follow up clinics. The accession numbers of the sequences obtained from the database are listed in S2 Table.

## Results

A total of 744 respiratory specimens (358, 260, and 126 for each season) positive for HRSV by RDT were collected during September 2012 through August 2015 at 18 pediatric outpatient clinics and hospitals in 17 prefectures in Japan (Table 2).

**Table 2:** Subgroup of HRSV divided by prefecture during the 2012–2013 through the 2014–2015 seasons.

Prefectures	2012–2013 season, No. (%)			2013–2014 season, No. (%)			2014–2015 season, No. (%)		
	HRSV-A	HRSV-B	Total	HRSV-A	HRSV-B	Total	HRSV-A	HRSV-B	Total
Hokkaido	–	–	–	–	–	–	11 (84.6)	2 (15.4)	13 (100)
Aomori	17 (94.4)	1 (5.6)	18 (100)	7 (70.0)	3 (30.0)	10 (100)	9 (81.8)	2 (18.2)	11 (100)
Chiba	7 (77.8)	2 (22.2)	9 (100)	3 (50.0)	3 (50.0)	6 (100)	–	–	–
Tokyo	5 (83.3)	1 (16.7%)	6 (100)	3 (50.0)	3 (50.0)	6 (100)	11 (84.6)	2 (15.4)	13 (100)
Kanagawa	5 (100.0)	0 (0.0)	5 (100)	17 (68.0)	8 (32.0)	25 (100)	–	–	–
Niigata	96 (73.8)	34 (26.2)	130 (100)	12 (27.9)	31 (72.1)	43 (100)	38 (100)	0	38 (100)
Shizuoka	2 (100.0)	0 (0.0)	2 (100)	2 (22.2)	7 (77.8)	9 (100)	6 (100)	0	6 (100)
Aichi	6 (85.7)	1 (14.3)	7 (100)	0	11 (100.0)	11 (100)	–	–	–
Mie	5 (100.0)	0 (0.0)	5 (100)	11 (100)	0	11 (100)	4 (100)	0	4 (100)
Shiga	–	–	–	4 (25.0)	12 (75.0)	16 (100)	4 (100)	0	4 (100)
Osaka	6 (60.0)	4 (40.0)	10 (100)	9 (81.8)	2 (18.2)	11 (100)	–	–	–
Hyogo	5 (71.4)	2 (28.6)	7 (100)	1 (20.0)	4 (80.0)	5 (100)	–	–	–
Kagawa	7 (61.5)	6 (38.5)	13 (100)	–	–	–	–	–	–
Yamaguchi	10 (90.9)	1 (9.1)	11 (100)	7 (38.9)	11 (61.1)	18 (100)	–	–	–
Fukuoka	18 (100.0)	0 (0.0)	18 (100)	–	–	–	–	–	–
Kumamoto	5 (100.0)	0 (0.0)	5 (100)	1 (5.6)	17 (94.4)	18 (100)	22 (95.7)	1 (4.3)	23 (100)
Okinawa	–	–	–	20 (100)	0	20 (100)	4 (80.0)	1 (20.0)	5 (100)
All	194 (78.9)	52 (21.1)	246 (100)	97 (46.4)	112 (53.6)	209 (100)	109 (93.2)	8 (6.8)	117 (100)

Note. “–”denotes sample collection not conducted

### Geographic distribution of HRSV subgroups

**Fig 1:**
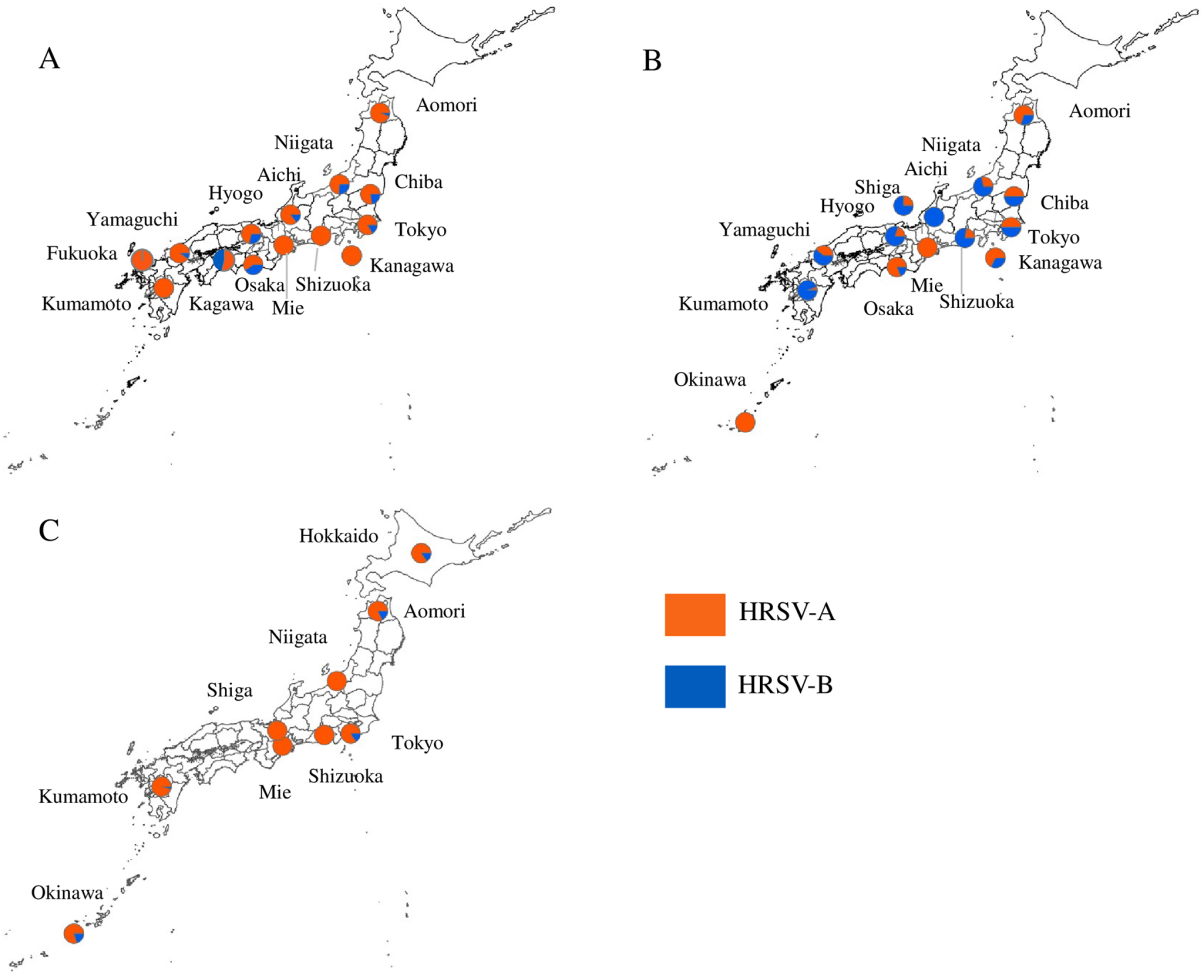
Geographic distribution of HRSV subgroups during 2012–2013 (A), 2013–2014 (B) and 2014–2015 (C) seasons in Japan.

Out of 744 samples, 572 (76.9%) were positive for HRSV by real-time PCR; 400 were HRSV-A and 172 HRSV-B. HRSV-A predominated during the 2012–2013 (78.9%) and 2014–2015 (93.2%) seasons, while similar levels of HRSV-A and -B circulation was recorded during the 2013–2014 (46.4% and 53.6%, respectively) (Table 2 and Fig 1) During the 2012–2013 season, HRSV-A constituted 60–100% of the positive cases in all of the 14 prefectures for which specimens were available. During the subsequent season, 7 of the 14 prefectures displayed predominance of HRSV-B (61.1–100%). In the 2015–2015 season, all of the 9 prefectures showed HRSV-A majority (81.9–100%). During the three seasons, a shift from HRSV-A to HRSV-B and then to HRSV-A was observed in 3 prefectures (Niigata, Shizuoka, and Kumamoto).

### Prevalence of HRSV genotypes

For 400 HRSV-A, sequences of the G protein’s HVR2 were obtained for 356 specimens, of these 242 (68%) were NA1 and 114 (32%) were ON1 by the phylogenetic tree analysis (Table 3). For 172 HRSV-B, sequences were successfully determined for 87 specimens; of these 60 (69%) were BA9 and 27 (31%) were BA10. Forty-four (11.0%) of 400 HRSV-A and 85 (49.4%) of 172 HRSV-B that were real-time PCR positive but genetic sequences not available were classified as untypable.

**Table 3:** Number of HRSV cases by genotype during the 2012–2013 and 2014–2015 seasons.

HRSV subgroup	HRSV genotype	No.(%) of positives			Total No. (%)
		2012–2013	2013–2014	2014–2015	
HRSV-A	Total	194 (100)	97 (100)	109 (100)	400(100)
	NA1	170 (87.6)	45 (46.4)	27 (24.8)	242 (60.5)
	ON1	8 (4.2)	39 (40.2)	67 (61.5)	114 (28.5)
	Untypable	16 (8.2)	13 (13.4)	15 (13.7)	44 (11.0)
HRSV-B	Total	52 (100)	112 (100)	8 (100)	172 (100)
	BA9	22 (42.3)	31 (27.7)	7 (87.5)	60 (34.9)
	BA10	13 (25.0)	14 (12.5)	0 (0.0)	27 (15.7)
	Untypable	17 (32.7)	67 (59.8)	1 (12.5)	85 (49.4)

**Fig 2:**
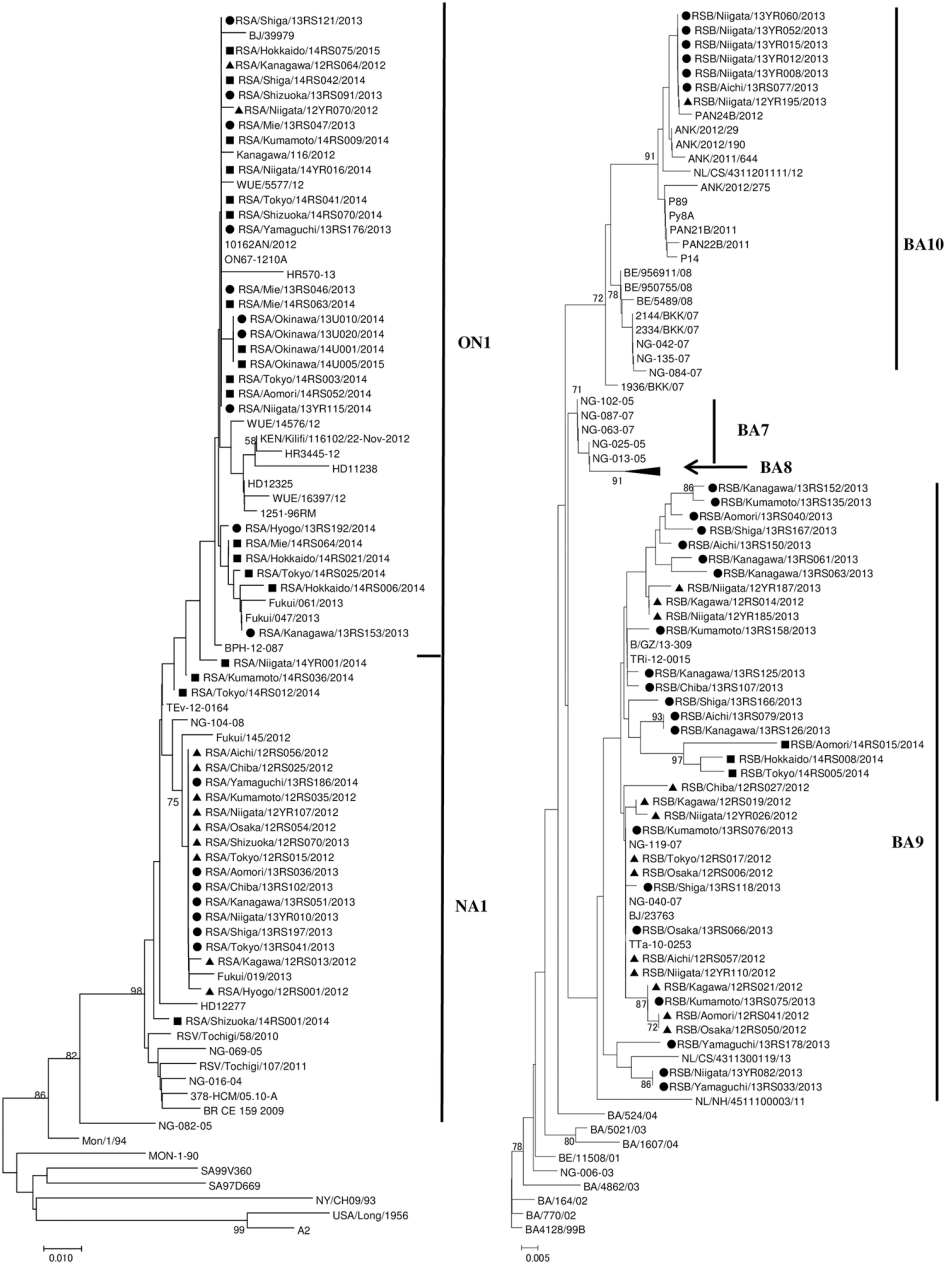
Phylogenetic trees of HRSV-A (A) and HRSV-B (B) strains. The tree was constructed by the neighbor-joining method using the Maximum Composite Likelihood for substitution model and complete deletion for gap or missing data treatment (MEGA, version 6). Bootstrap value was determined for 1000 iterations. Only values greater than 70% are shown. Strains detected during the 2012–2013 season are indicated by closed triangle (▲), those during the 2013–2014 season by closed circle (●), and those during the 2014–2015 season by closed square (■). Reference sequences of HRSV-A and HRS-B strains downloaded from GenBank (S2 Table) were compared with strains detected in this study (S1 Table).

Phylogenetic tree analysis revealed that the majority of the HRSV-A NA1 strains from the 2012–2013 and 2013–2014 seasons clustered together sharing high bootstrap value (Fig 2). While those from the 2014–2015 season did not tightly cluster together. The ON1 strains for the three seasons fell in one cluster. The average p-distance within NA1 and ON1 in the cluster was 0.019 and 0.013, respectively, while the mean distance between the two was 0.039.

The HRSV-B specimens belonged to two genotypes, BA9 and BA10 (Fig 2). The former accommodated the majority of the Japanese specimens from the three seasons, which were more diverse than the specimens belonging to the BA10. The average p-distances in the cluster were 0.023 and 0.015 within BA9 and BA10 sequences, respectively. The p-distance value between BA9 and BA10 was 0.054 showing the two are distinct clades.

### Monthly and geographic distribution of the HRSV genotypes in Japan

**Fig 3:**
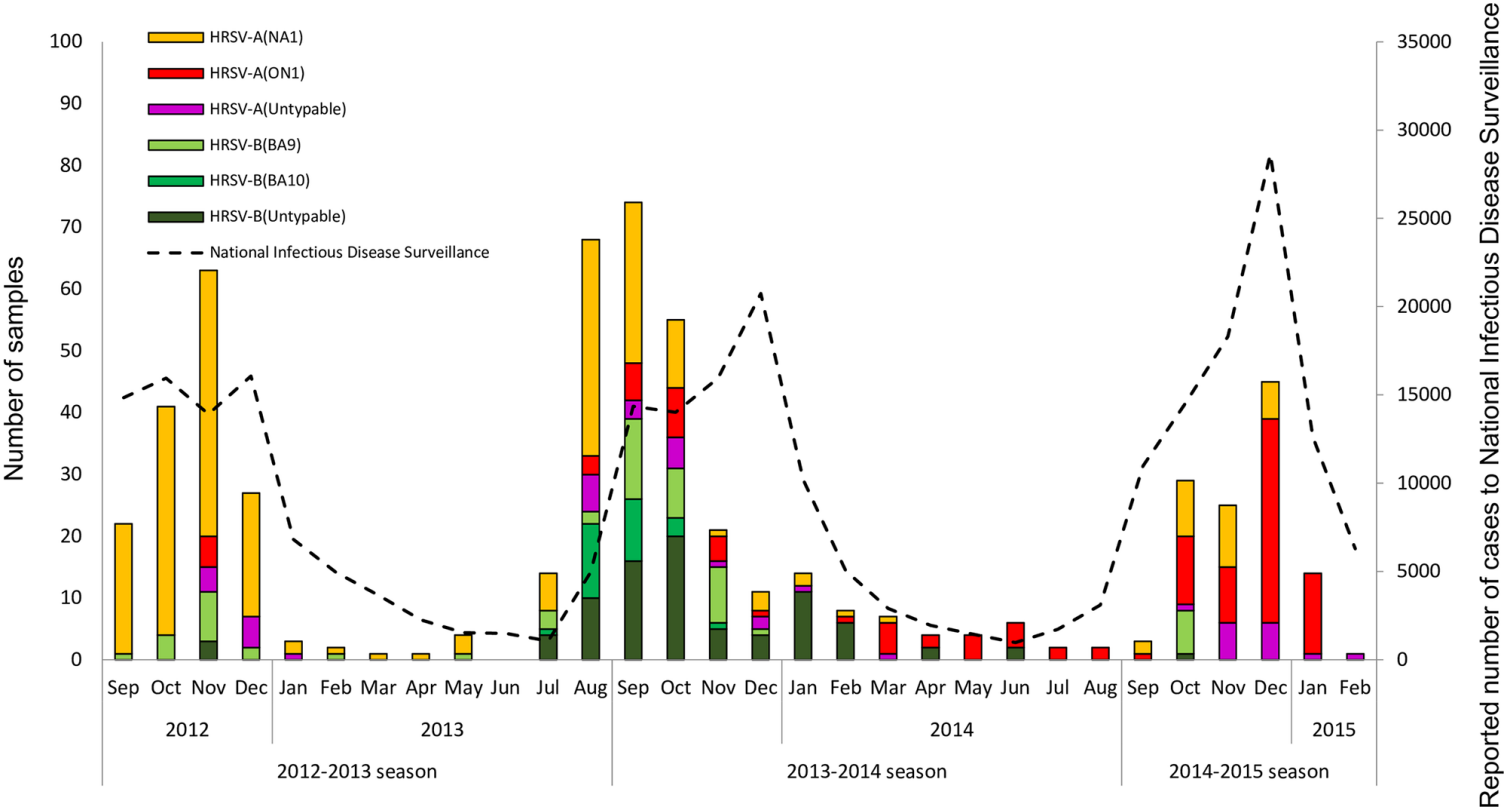
Monthly distribution of HRSV cases and genotypes in Japan. The bar graph shows the number of HRSV cases by genotype detected in this study and the line graph represents the number of HRSV cases reported to National Infectious Disease Surveillance.

**Fig 4:**
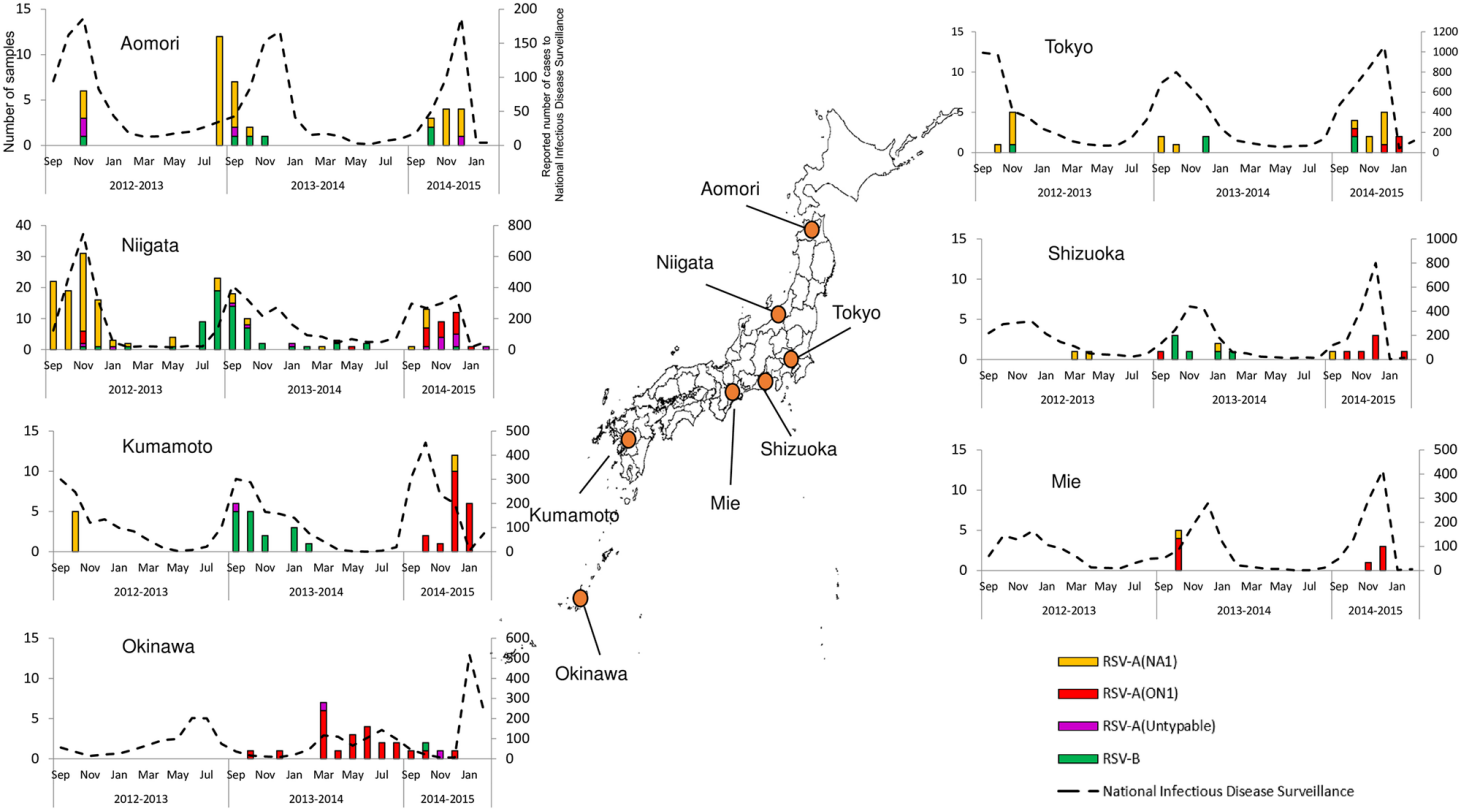
Monthly distribution of HRSV cases by location during the three seasons. Data is shown for the six prefectures where specimens were collected during three seasons besides Okinawa. Left y-axis shows the number of HRSV specimens by genotype detected in this study and the right y-axis represents the number of HRSV cases reported to National Infectious Disease Surveillance in each prefecture.

HRSV activity in Japan displayed clear epidemic period that peaked during the winter and continued year round albeit a lower rates during the inter-epidemic period. During 2012–2013 season, the number of laboratory HRSV-confirmed cases peaked in November 2012 and NA1 was the predominant genotype (Fig 3). The ON1 genotype was first detected in Kanagawa (RSA/Kanagawa/12RS064/2012) and Niigata (RSA/Niigata/12YR070/2012) in November 2012 in this study (Fig 3, S1 Table). According to the National Infectious Disease Surveillance, HRSV cases peaked in October to December 2012, consistent with our data. During the 2013–2014 season, HRSV activity peaked in September 2013 and continued throughout the year albeit at low rate during the spring and fall. Interestingly, in the national data the peak of HRSV activity was observed in December, i.e. two months after the peak observed in our laboratory based surveillance. This could be attributed to the drop in enthusiasm of clinicians to submit specimens as the season progressed and in some cases the dwindling supplies of viral transport media in the clinic as cases increased resulted in less sampling. In 2013–2014 season, the NA1 genotype continued to circulate. The BA10 genotype was first detected in July 2013 (2012–2013 season) and lasted only for five months until November 2013 (2013–2014 season) (Fig 3, S1 Table). The incidence of the ON1 genotype increased compared to the previous season but it remained the minor strain during the 2013–2014 season except for Okinawa (Fig 4 and S3 Table). During the 2014–2015 season, our laboratory-confirmed cases peaked in December 2014, during which we observed a shift to ON1 as the main circulating strain (Fig 3). Six out of 8 prefectures had a shift of predominant genotype from NA1 to ON1 (S3 Table). Simultaneously, the National Infectious Disease Surveillance recorded approximately 30,000 cases nationwide in December 2014, the highest number of HRSV cases ever since the start of the national HRSV surveillance in 2003[35].

At individual prefecture level where data are available for three seasons, prefectures such as Niigata or Kumamoto showed HRSV epidemic period during July to January with peaks around September to December, which generally coincided with the data from the National Infectious Diseases Surveillance (Fig 4). Other areas where sampling was biased and positives were less (e.g. Mie) did not form clear epidemic period but the National Infectious Disease data supplemented similar peak periods. The reason for the poor sampling in these locations was mainly due to limited time of the clinicians to spare for the study, e.g. obtaining informed consent from patients, collecting specimens and necessary clinical information from the medical records. Okinawa, southernmost islands with subtropical climate in Japan, had a different timing of HRSV activity, which was observed from March to October with peaks in July. Therefore, the inter-epidemic HRSV activity observed in the cumulative activity plots can be largely attributed to Okinawa. The ON1 genotype represented the majority of HRSV circulating in Okinawa in 2014 prior to becoming the dominant strain nationwide during 2014–2015 season (Fig 4).

### Evaluation of hospitalization risk by HRSV genotype

There were 66 hospitalized and 383 non-hospitalized patients during the study period. Patient characteristics and HRSV genotypes were compared between hospitalized and non-hospitalized patients using uni-variate analysis. Age distribution of patients who were hospitalized and non-hospitalized did not show difference, 11.7 ± 10. 2 months, and 15.5 ± 10.0 months old, respectively (P = 0.40) (Table 4). The hospitalization rate was significantly higher in children less than 6 months, prematurely born and/or having low birth weight, with underling conditions, and those infected with the ON1 genotype in univariate analysis (Table 4). Adjusted for all other factors, the hospitalization risk was found to be 6.9-fold higher for the ON1-infected group compared with the reference NA1 group (*P*< 0.001, odds ratio 6.92 [95% confidence interval, 3.45–13.90]) using logistic regression analysis (Table 5).

**Table 4:** Univariate analysis of the baseline characteristics and HRSV genotypes of hospitalized and non-hospitalized patients.

	No. (%) of HRSV cases		*P* value
	Hospitalized N = 66	Non-hospitalized N = 383	
Age			
Average±standard deviation(month)	11.7 ± 10.2	15.5 ± 10.0	0.40
< 6 months	27 (30.3%)	62 (69.7%)	
≥ 6 months	38 (10.7%)	317 (89.3%)	<0.001
Sex			
Male	43 (18.3%)	192 (81.7%)	
Female	23 (11.7%)	173 (88.3%)	0.08
Prematurity and/or low birth weight ^a^			
Yes	13 (34.2%)	25 (65.8%)	
No	53 (12.9%)	358 (87.1%)	<0.001
Underlying conditions ^b^			
Yes	13 (61.9%)	8 (38.1%)	
No	53 (12.4%)	375 (87.6%)	<0.001
Genotype			
NA1	16 (6.6%)	227 (93.4%)	
ON1	42 (35.6%)	76 (64.4%)	
BA9	7 (11.5%)	54 (88.5%)	
BA10	1 (3.7%)	26 (96.3%)	<0.001

Missing data exists in some categories.

^a^ Prematurity was defined as gestation week <36 weeks, and low birth weight was <2500g.

^b^ Underlying condition includes congenital heart diseases, congenital chronic lung diseases, immune deficiencies, Down Syndrome, or asthma.

**Table 5:** Hospitalization risk by HRSV genotype using multivariate analysis.

Genotype of HRSV	Odds ratio (95% CI)	*P* value
NA1	Reference	
ON1	6.92 (3.45–13.90)	<0.001 ^a^
BA9	1.56 (0.55–4.43)	0.443
BA10	0.66 (0.08–5.35)	0.693

CI: confidence interval

^a^ Logistic regression analysis was performed adjusted for age (< 6 or ≥ 6 months), prematurity (gestation week <36 weeks) and/or low weight birth (< 2500g), and underlying conditions.

## Discussion

The National Infectious Disease Surveillance in Japan currently only reports HRSV cases mainly diagnosed by RDTs but does not investigate the genetic diversity of the virus and its association with disease prevalence. There are a few reports on the genetic diversity and the circulating genotypes of HRSV in Japan; however, the sampling locations of these studies were limited [36–38]. This study represents the first molecular epidemiology study investigating the diversity of HRSV during three successive seasons, covering 17 prefectures across Japan.

In this study, we observed a change in the prevailing HRSV subgroup by which the dominant subgroup alternated between HRSV-A and -B during the successive seasons. The predominant subgroup tended to be the same across all prefectures especially when HRSV-A is predominated. We could not compare our results with others because no study has been reported to investigate the diversity and geographic distribution of HRSV during the same period in Japan. We have previously observed a similar pattern in one prefecture in Japan over a period of 9 seasons, between 2001 through 2010 [16]. This alternate pattern of HRSV-A and -B seasons has been also observed in other countries including Finland[39], China [40–42], the Philippines [43,44], Argentina [45], Malaysia [46], and Senegal [47]. The alternation between the HRSV subgroups in subsequent seasons can be attributed to the variation in the immunity of the host population.

We observed dominant strain of HRSV-A changed from NA1 to ON1 during the study period. NA1 was derived from GA2 and we first detected in 2004 in Niigata, Japan [11]. Since then, NA1 was the predominant HRSV-A genotype circulating in Japan for nearly 10 years [36,48]. It was reported in various countries worldwide [12,28,33,42,43,46,49–56]. Recently Trento et al. proposed a new definition for clade classification to set p-distance less than 0.049 within a group. According to their analysis all of the NA1, NA2, NA4, and ON1 genotypes were reclassified into the GA2 genotype [33]. We also used this definition in this study and the p-distance showed that NA1 and ON1 could be classified into a single clade (p-distance, 0.039). However, we retained NA1 and ON1 as independent clades in this study to keep consistency from the previous reports by the reason for an unique 72 nucleotide duplication observed in ON1 [12,37,54,56–60].

The ON1, with a 72 nucleotide duplication in the C-terminal region of the G protein, was first detected in hospitalized children in Ontario, Canada in December 2010 [12]. Since then the genotype was detected various parts of the world and replaced NA1 [14,18,25,28,37,41,42,44–47,56–59,61–67]. In Japan, the ON1 strain was first detected in 2012 (this study and Tsukagoshi et al)[37] but it did not prevail until winter during 2014–2015 season as was shown in this study. In neighboring China, ON1 was initially detected in February 2011 and became the dominant HRSV-A genotype in December 2013[42]. Other countries, such as Vietnam, the Philippines, Italy and Kenya, reported that the shift from NA1 to ON1 predominance occurred during the 2012–2013 season, almost one to two years earlier than in Japan and mainland China[22,44,56,67]. Evolutionary analysis of the ON1 strain suggests that this genotype might have emerged from the Americas, specifically in Panama around 2010 [45]. Our data and those from previous studies suggest that the ON1 rapidly spread worldwide after its emergence [25] and the time needed to replace the previous RSV-A genotype NA1 was 2 to 4 years [22,42,44]. A report from Kenya showed that the replacement rate was quicker than the previous genotypes: GA2 took almost 7 years to replace GA5 [22]. The rapid replacement caused by ON1was largely attributed to the 72 nucleotide duplication and genetic diversity of the ectodomain of G protein [22,25,36,68]. However, there are conflicting views as to the impact of the variability of the ectodomain of the G protein on its reaction with antibodies. Trento et al. reported genetic diversity of G protein and temporal genotype dominance could not be directly related to antigenic changes [33]. Further studies comparing the genetic diversity and antibody reaction are needed to elucidate the molecular factors that might have contributed to its successful spread.

The emergence of ON1 as the predominant strain circulating in Japan during the 2014–2015 season was associated with a record high number of HRSV cases being reported to National Infectious Disease Surveillance compared to last 10 seasons[35]. The level of surveillance was stable in terms of number of sentinel sites, prefectures, case definition, and testing criteria for both in this study and the National Infectious Disease Surveillance during the study period. Thus the emergence of ON1 could be the reason for a large outbreak in Japan during 2014–2015 season. Similarly, large outbreaks occurred with the emergence of NA1 in Niigata prefecture in Japan between 2005 and 2007 [11]. In the contrary, epidemic size did not seemingly increased in other countries, such as China and Kenya where they have nationwide or continuous surveillance over the years [22,42]. Association between new genotypes and the epidemic size should be further investigated.

Similar to HRSV-A, HRSV-B has evolved into multiple genotypes over the past decade[17]. The BA genotype, with a 60-nucleotide duplication in the G protein, was first detected in Argentina in 1999 and spread throughout the world [17]. In Japan, we first reported the BA genotype in 2002–2003[69]. The BA strain diversified over time into 10 clusters, including BA7-BA10, which we discovered in Japan [16]. Similar strains belonging to BA10 which we found in this study was reported not only from Japan but also from Germany, Belgium, Netherlands, Turkey, Panama, and Paraguay (S2 Table)[70]. This BA10 was short lived and circulated only for several months. The small number of sequences belonging to this clade in the database curtails the precise tracking of its origin.

Our study revealed that in Okinawa, southernmost island of Japan, HRSV epidemic occurs during March to September or the inter-epidemic period of Japan’s main islands. Accordingly, the shift to ON1 dominance occurred earlier than the rest of the country. Similar observations were also noted for influenza when the emergence of amantadine-resistant H3N2 viruses in Okinawa occurred a few months ahead of their spread to the main islands [71]. The inter-epidemic activity of HRSV in Okinawa was strongly associated with higher temperature (>28°C) and higher relative humidity (>79.0%) as reported elsewhere [72]. A similar correlation to hot and wet weather was also demonstrated in Hong Kong, with similar climate to Okinawa, and where HRSV follows a dual peak pattern during March-April and July to October [73,74]. Prior to community circulation in Okinawa, ON1 were observed in the Philippines in 2013 and China in 2014[42,44]. Although limited data are available, ON1 in Okinawa were seemingly derived from other countries in Asia and eventually caused a big outbreak several months later in the main islands of Japan in 2014. Further phylogeographic studies are warranted to confirm whether the Okinawa is the intersection between other Asian countries and Japan, but close monitoring of HRSV activity and the circulating genotypes in Okinawa may allow early prediction of the subsequent epidemic season in Japan.

We demonstrated that ON1 was associated with significantly higher hospitalization risk than NA1 after adjustment for various background factors. Numerous clinical factors are related to increased risk for hospitalization with RSV infection, such as < 6 month old, male, preterm gestation week, low birthweight, congenital heart disease, bronchopulmonary dysplasia, history of atopy (including asthma) [75–77]. In this study we divided age group at 6 months old because the frequency of hospitalization was higher (30.3%) as opposed to the division at 12 months old (20.3%). We grouped asthma together with other underlying conditions since the frequency of hospitalization under children with asthma (5/9, 55.6%) was not different from those with the other underlying conditions (8/12, 66.7%, P = 0.673). Apart from host factors, we found a viral factor, infection with ON1 genotype, was associated with the increased risk for hospitalization. So far, conflicting results were reported on the varying disease severity in correlation with the HRSV genotype. Otiento et al. found that patients infected with ON1 were twice more likely to be unable to eat than those with GA2 (including NA1) infections[22]. Nonetheless, the proportions of patients with very severe pneumonia were similar for both genotypes[22]. Yoshihara et al. reported hospitalization incidence was significantly increased after the emergence of ON1, and risk of lower respiratory tract infection was 2.26 (95% CI: 1.37–3.72) times higher, and radiologically confirmed pneumonia was 1.98 (95% CI: 1.01–3.87) times greater in ON1 compared to NA1 [78]. A report from Brazil also noted an increase of intensive care unit admissions and need for mechanical ventilation associated with ON1genotype [79]. In contrast, reports from Italy, the Philippines, South Africa, Spain found no evidence of clinical differences in disease severity in correlation to genotype [30,56,80,81].

In this study we used RDT for screening of HRSV because it is commonly used in clinical settings in Japan. The RDTs are covered by health insurance in Japan for the following conditions; children under 1 year old at outpatient clinics, those who need hospital admission (without clear age definition), and children eligible for palivizumab administration. Choice of which RDT products to use was up to clinicians but we additionally supplied one of the RDT, Quick-navi Flu+RSV to avoid potential age bias in sampling for children ≥1 years old at outpatient clinics who were not covered by the health insurance. The sensitivity and specificity of Quick-navi Flu+RSV was 92.5% and 76.8% respectively (N = 229 cases) against real time PCR in the other study with the same clinicians, which demonstrated enough sensitivity for the screening purpose. We believe the difference of sensitivity for each RDT on subgroup A and B did not affect the detection of HRSV. One of the RDT, Quick-navi Flu+RSV, showed the similar sensitivity for HRSV-A 87.5% (N = 56) and that of HRSV-B 97.2% (N = 36) against subgrouping real time PCR. We could not calculate specificity for subgroups because RDT can detect only generic HRSV. In this study, identification rate of HRSV-B genotype (50.6%) against real time PCR HRSV-B were low compared to better identification of HRSV-A genotype (89.0%), with the reasons remained unknown. The other study in China also reported lower genotype identification for HRSV-B (30.1%) compared to HRSV-A (41.1%) [51]. Secondary structure formation during the reverse transcription or nucleotide changes specific to HRSV-B may result in the lower identification of genotypes.

This study had a number of limitations. First, irregular participation of sampling locations during the study period hindered comparative analysis of the circulation patterns of HRSV in different prefectures. The clinicians in our group mainly belonged to the Society of Ambulatory and General Pediatrics of Japan located in various parts of Japan, and we tried to enroll clinicians located at representative major geographic boundaries covering north to south. Of the 17 prefectures included in the study, only 6 prefectures had continuous sampling throughout three seasons while the other prefectures participated during only one or two years. In addition, only one site from each prefecture was included in the study, and variable number of specimens were submitted and some had few samples only. Despite these limitations, we believe that the collective data from all sites provides a close representation of HRSV circulation across Japan. The gap of peaks between our sampling and the National Infectious Disease surveillance was noted in 2013–2014 seasons. This was addressed in the following season by ensuring that clinics have enough supplies of transport media and they could collect samples in a timely manner.

In this study we only used partial G protein HVR2 for genetic analysis. However longer sequences (e.g. full lengths of G protein) are useful to elaborate more phylogenetic signal and better resolution in the evolutionary analysis [7,17,27,33,51,81,82] to understand the effect of any genetic changes on fitness, virulence and transmissibility [22]. We intend to continue investigating country wide patterns and genetic variety of HRSV and its correlation with clinical severity.

## Supporting information

S1 Table List of HRSV specimens sequenced in this study. (XLSX)

S2 Table Reference sequences of HRSV-A and B used for phylogenetic tree analysis. (XLSX)

S3 Table Number of HRSV genotypes detected in each prefecture between the 2012–2013 through the 2014–2015 seasons. (DOCX)
