# Supplementary material for: Molecular epidemiology of human respiratory syncytial virus among children in Japan during three seasons and hospitalization risk of genotype ON1
Source: PLoS One. 2018 Jan 29;13(1):e0192085. doi: 10.1371/journal.pone.0192085 (PMC5788364; doi:10.1371/journal.pone.0192085)
Supplement: S3 Table — (DOCX) [file pone.0192085.s003.docx]

S3 Table. Number of HRSV genotypes detected in each prefecture between the 2012-2013 and the 2014-2015 seasons.

| Prefectures |  | | 2012-2013 season, No. | | |  |  | | 2013-2014 season, No. | | |  |  | | 2014-2015 season, No. | | |
| --- | --- | --- | --- | --- | --- | --- | --- | --- | --- | --- | --- | --- | --- | --- | --- | --- | --- |
|  | NA1 | ON1 | | BA9 | BA10 |  | NA1 | ON1 | | BA9 | BA10 |  | NA1 | ON1 | | BA9 | BA10 |
| Hokkaido | － | － | | － | － |  | － | － | | － | － |  | 5 | 6 | | 2 | 0 |
| Aomori | 15 | 0 | | 1 | 0 |  | 6 | 0 | | 1 | 0 |  | 5 | 3 | | 2 | 0 |
| Chiba | 6 | 0 | | 2 | 0 |  | 1 | 2 | | 2 |  |  | － | － | | － | － |
| Tokyo | 5 | 0 | | 1 | 0 |  | 3 | 0 | | 0 | 0 |  | 7 | 4 | | 2 | 0 |
| Kanagawa | 3 | 1 | | 0 | 0 |  | 12 | 3 | | 7 | 0 |  | － | － | | － | － |
| Niigata | 90 | 4 | | 9 | 13 |  | 6 | 2 | | 2 | 13 |  | 7 | 19 | | 0 | 0 |
| Shizuoka | 2 | 0 | | 0 | 0 |  | 1 | 1 | | 0 | 0 |  | 1 | 5 | | 0 | 0 |
| Aichi | 5 | 0 | | 1 | 0 |  | 0 | 0 | | 3 | 1 |  | － | － | | － | － |
| Mie | 2 | 3 | | 0 | 0 |  | 2 | 8 | | 0 | 0 |  | 0 | 4 | | 0 | 0 |
| Shiga | － | － | | － | － |  | 1 | 2 | | 6 | 0 |  | 0 | 4 | | 0 | 0 |
| Osaka | 2 | 0 | | 3 | 0 |  | 8 | 0 | | 1 | 0 |  | － | － | | － | － |
| Hyogo | 5 | 0 | | 0 | 0 |  | 1 | 0 | | 0 | 0 |  | － | － | | － | － |
| Kagawa | 7 | 0 | | 4 | 0 |  | － | － | | － | － |  | － | － | | － | － |
| Yamaguchi | 10 | 0 | | 1 | 0 |  | 4 | 2 | | 2 | 0 |  | － | － | | － | － |
| Fukuoka | 13 | 0 | | 0 | 0 |  | － | － | | － | － |  | － | － | | － | － |
| Kumamoto | 5 | 0 | | 0 | 0 |  | 0 | 0 | | 7 | 0 |  | 2 | 19 | | 0 | 0 |
| Okinawa | － | － | | － | － |  | 0 | 19 | | 0 | 0 |  | 0 | 3 | | 1 | 0 |
|  |  |  | |  |  |  |  |  | |  |  |  |  |  | |  |  |
| All | 170 | 8 | | 22 | 13 |  | 45 | 39 | | 31 | 14 |  | 27 | 67 | | 7 | 0 |

Note. “－”denotes sample collection not conducted
